# A newborn with type 1 Bartter syndrome: challenges in the treatment and development during 30 months follow-up—a case report

**DOI:** 10.3389/fped.2026.1772606

**Published:** 2026-03-10

**Authors:** Justyna Czubilińska-Łada, Anna Szymańska, Anna Sienko, Andrzej Badeński, Jakub Behrendt, Maria Szczepanska

**Affiliations:** 1 Department of Neonatal Intensive Care and Neonatal Pathology, Faculty of Medical Sciences in Zabrze, Medical University of Silesia in Katowice, Zabrze, Poland; 2 Department of Pediatrics, Faculty of Medical Sciences in Zabrze, Medical University of Silesia in Katowice, Zabrze, Poland

**Keywords:** Bartter syndrome type I, celecoxib, chronic kidne disease, nephrocalcinosis, newborn

## Abstract

Type I Bartter syndrome is a rare autosomal recessive tubulopathy resulting from mutations in the SLC12A1 gene, leading to defective sodium–chloride reabsorption in the thick ascending limb of the loop of Henle. Affected neonates typically present with profound fluid and electrolyte disturbances, polyuria, and metabolic derangements. Early diagnosis and individualized management are crucial to prevent life-threatening complications and support appropriate growth and development. We report the case of a male preterm infant born at 33 weeks’ gestation following a pregnancy complicated by severe polyhydramnios requiring multiple amnioreductions. At 2 weeks of age, he was admitted with severe dehydration, acute kidney injury (creatinine 205 µmol/L; eGFR 8 mL/min/1.73m^2^), marked polyuria (300 mL/kg/day), and significant electrolyte abnormalities, including hyponatremia, hypokalemia, hypochloremia, and metabolic acidosis. During the first week of hospitalization, he developed necrotizing enterocolitis. Bartter syndrome was suspected based on the biochemical profile, perinatal history, and persistent electrolyte imbalance, and subsequently confirmed by identifying a pathogenic SLC12A1 variant (NM_001184832:c.1327G>A). Management required exceptionally high fluid volumes (280–310 mL/kg/day), intensive sodium and potassium supplementation, and gradual transition from parenteral to enteral nutrition. Following expert consultation, celecoxib was introduced (2.5 mg/kg twice daily), permitting stabilization of electrolyte homeostasis and discontinuation of parenteral nutrition by day 61 of life. Throughout hospitalization, complications included catheter-related inflammation, inferior vena cava thrombosis, anemia requiring transfusion, and stage II intraventricular hemorrhage. Serial renal ultrasonography demonstrated persistent nephrocalcinosis. During 30 months of follow-up, the patient exhibited normal neurodevelopmental progress but persistent challenges in growth, requiring endocrinology supervision. Laboratory parameters generally remained stable except for periodic hypercalciuria (Ca/Crea >0.3). Episodes of intercurrent infections led to rapid electrolyte deterioration, necessitating intensified monitoring and supplementation. Celecoxib therapy remained essential, with unsuccessful attempts at dose reduction. Nephrocalcinosis persisted without deterioration in renal function. This case highlights that early diagnosis of type I Bartter syndrome enables timely targeted therapy but achieving stable fluid, electrolyte, and nutritional status remains challenging. Long-term management requires multidisciplinary care, vigilant monitoring during intercurrent illnesses, and individualized adjustments in pharmacologic and nutritional therapy. This report contributes valuable longitudinal insight into the complexities of managing neonatal-onset Bartter syndrome.

## Introduction

**Figure 1:**
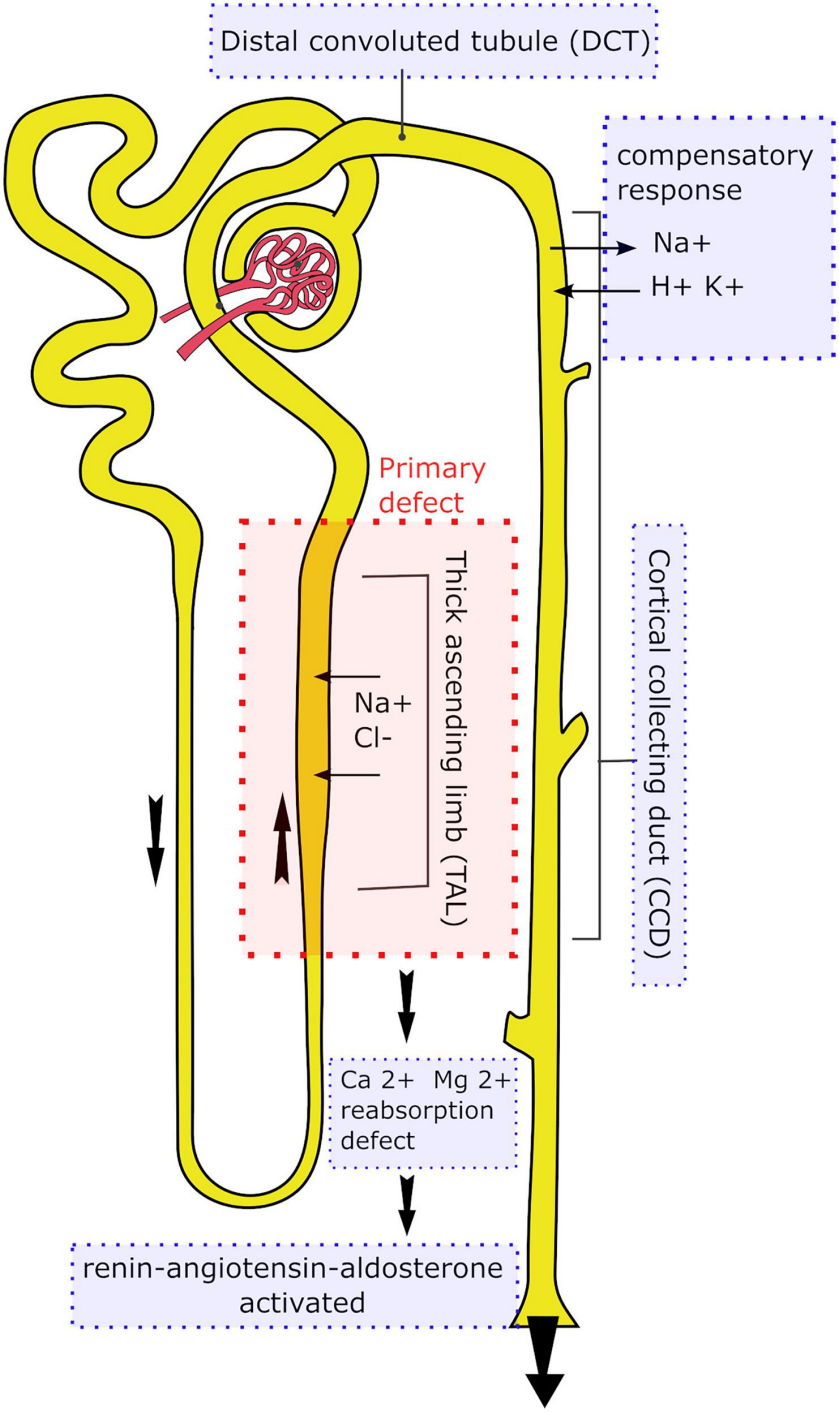
Primary and secondary pathophysiological defects within the nephron in Bartter syndrome.

Bartter's syndrome (BS) is a rare group of hereditary tubulopathies caused by impaired salt reabsorption primarily in the thick ascending limb (TAL) of the loop of Henle, with secondary functional disturbances in the distal convoluted tubule (DCT) and the cortical collecting duct (CCD). Five types of Bartter syndrome (I–V) have been described, differing in their genetic background, all of which result from defects in genes encoding sodium–chloride transport mechanisms in the TAL. Sodium transporters in the DCT and CCD are structurally normal, but their function becomes altered as a compensatory response (1–3) [Figure 1].

Disrupted sodium transport in the thick ascending limb (TAL) leads to a substantial loss of sodium ions, as well as chloride ions that are normally transported together. Moreover, the kidney's ability to concentrate urine becomes impaired, and the reabsorption of magnesium and calcium ions is reduced, as these ions are normally reabsorbed passively via the paracellular pathway driven by the electrochemical gradient generated by active salt transport. This defect activates the renin–angiotensin–aldosterone system. To compensate for sodium loss, ion transport mechanisms in DCT and CCD become upregulated, allowing partial reclamation of sodium at the expense of increased potassium and hydrogen ion secretion. As a consequence, patients present with polyuria, hyponatremia, hypokalemia, hypochloremic metabolic alkalosis, hypercalciuria, and secondary hyperreninemic hyperaldosteronism with normal or low blood pressure (4–8).

An additional important mechanism in the pathophysiology of Bartter syndrome involves impaired chloride delivery to the macula densa, which disrupts tubuloglomerular feedback. This leads to activation of cyclooxygenase and excessive production of prostaglandin E₂, further stimulating renin release and aldosterone synthesis, thus exacerbating polyuria, hypovolemia, and symptoms such as fever or gastrointestinal disturbances (9).

Among the five currently recognized types of Bartter syndrome, several may present during infancy, including types 1, 2, 4, and 5. Most forms, however, characteristically manifest prenatally and are frequently associated with severe polyhydramnios requiring repeated amnioreductions during pregnancy (2, 10, 11).

## Case report

### Admission

A 2-week-old male neonate was transferred to the Department of Neonatal Intensive Care and Neonatal Pathology in Zabrze from a regional hospital due to severe dehydration and acute kidney injury accompanied by profound acid–base and electrolyte disturbances. The infant was born at 33 weeks' gestation via cesarean section, performed due to a history of previous cesarean delivery and significant polyhydramnios. The pregnancy was complicated by recurrent excessive amniotic fluid, necessitating three amnioreduction procedures. The family history was non-contributory. At birth, the neonate presented with a weight of 1785 g, body length of 46 cm, head circumference of 31.5 cm and chest circumference of 27 cm. He did not require respiratory support in the immediate postnatal period.

Upon admission, the infant appeared severely dehydrated and cachectic, with a 9% reduction in weight compared to birth weight. Despite preserved respiratory and circulatory stability, laboratory tests revealed several critical abnormalities. The patient exhibited marked polyuria (300 mL/kg/day) and acute kidney injury, with a peak serum creatinine concentration of 205 µmol/L and an estimated glomerular filtration rate (eGFR) of 8 mL/min/1.73m^2^. Additionally, significant metabolic acidosis, hyponatremia (lowest sodium level 121.1 mmol/L), hypokalemia (lowest potassium level 2.64 mmol/L) and hypochloremia (lowest chloride level 90.6 mmol/L) were noted. Blood pressure values remained within the normal range.

### Necrotizing enterocolitis

At the end of the first week of hospitalization, the patient's clinical condition deteriorated markedly. In addition to the previously observed abnormalities, new gastrointestinal symptoms emerged, including abdominal distension and signs of discomfort. Abdominal ultrasonography revealed pneumatosis intestinalis and the presence of gas within the portal venous system, findings consistent with a diagnosis of necrotizing enterocolitis (NEC). Potential contributing factors to the development of NEC in this patient included prematurity as well as severe nutritional disturbances leading to malnutrition and significant renal failure.

Enteral feeding was immediately discontinued, and broad-spectrum antimicrobial therapy with vancomycin and meropenem (administered in doses adjusted for renal impairment) was initiated. Over the following days, gastrointestinal symptoms gradually improved. Seven days after the onset of NEC, cautious reintroduction of enteral feeding was undertaken, with gradual advancement of feeding volumes. After a total of 20 days of therapy, antibiotics were discontinued.

### Diagnosis—Bartter syndrome

Following consultation with a pediatric nephrologist, Bartter syndrome was suspected based on the patient's clinical presentation, biochemical profile and perinatal history. Subsequent molecular testing confirmed the diagnosis, identifying a pathogenic variant (NM_001184832:c.1327G>A) in the *SLC12A1* gene, consistent with type I Bartter syndrome.

The diagnosis was further supported by characteristic biochemical findings, including markedly elevated plasma renin activity (498 ng/mL/h), elevated aldosterone levels (>1,000 pg/mL), and decreased urinary chloride concentration (64.5 mmol/L), consistent with the typical biochemical profile observed in Bartter syndrome. These laboratory abnormalities reflect chronic salt-wasting and secondary activation of the renin–angiotensin–aldosterone system, which are hallmarks of the disease.

Management focused on optimizing fluid and electrolyte balance. The patient's fluid and electrolyte requirements were substantially increased to achieve euvolemia and correction of electrolyte disturbances. Clinical stabilization was accompanied by a gradual decline in serum creatinine levels (to 52 µmol/L) and a shift in acid–base status toward metabolic alkalosis, characteristic of Bartter syndrome. Renal ultrasonography demonstrated loss of corticomedullary differentiation and features of nephrocalcinosis.

Total fluid requirements ranged from 280 to 310 mL/kg/day. Of this, approximately 160–170 mL/kg/day consisted of enteral feeding with an infant milk formula, which was well tolerated; the remaining volume was administered via a central venous catheter. Parenteral electrolyte supplementation included sodium and potassium, titrated to maintain serum concentrations at the lower limit of the normal range. At maximum, the patient received 13 mmol/kg/day of sodium and 8 mmol/kg/day of potassium.

### Introduction of celecoxib treatment

Nonsteroidal anti-inflammatory drugs (NSAIDs), including celecoxib, are commonly used in the treatment of Bartter syndrome to inhibit cyclooxygenase activity, thereby reducing excessive prostaglandin *E*_2_ production and subsequent activation of the renin–angiotensin–aldosterone system. In view of the patient's history of necrotizing enterocolitis, celecoxib was selected because of its more favorable gastrointestinal safety profile and its wide clinical availability. Due to our inexperience with Bartter syndrome, we sought expert guidance and contacted Prof. Detlef Bokenhauer from the Department of Paediatric Nephrology, UZ Leuven, and Prof. Sally Hulton from Birmingham Children's Hospital (12, 13). We received valuable recommendations regarding the use of celecoxib and the overall management of children with Bartter syndrome.

The experts advised initiating celecoxib at a dose of 2–5 mg/kg twice daily; our patient was started on an initial dose of 2.5 mg/kg twice daily. To protect the gastric mucosa, a proton pump inhibitor was co-administered. Additionally, the guidance included gradual reduction of parenteral fluid administration, preferential advancement of enteral feeding via a gastric tube, and tapering of intravenous sodium and potassium supplementation in favor of oral administration.

These measures allowed for progressive stabilization of fluid and electrolyte balance and enabled discontinuation of parenteral nutrition by day 61 of life.

### Further treatment and observation

During the fifth week of treatment, a marked increase in inflammatory markers was observed, most likely associated with a catheter-related infection, although blood cultures and central line tip cultures remained sterile. The patient was treated with linezolid, resulting in normalization of inflammatory parameters. In the seventh week, color Doppler ultrasonography revealed a small thrombus in the inferior vena cava originating from the central catheter, with preserved flow. Attempts at thrombolysis using a plasminogen activator were unsuccessful, and therapeutic low-molecular-weight heparin was continued.

Renal ultrasonography was performed regularly throughout hospitalization. Blurring of corticomedullary differentiation and multiple hyperechoic foci were noted, suggestive of early stages nephrocalcinosis.

### Other problems

During regular ultrasound examinations, other symptoms were described. A small amount of concentrated bile was observed in the gallbladder, prompting the initiation of ursodeoxycholic acid therapy. Moreover, bilateral second-degree intraventricular hemorrhage was diagnosed. Due to anemia, the patient received two transfusions of red blood cell concentrate and was treated with iron supplementation, hematopoietic vitamins, and erythropoietin.

### Stabilisation and discharge home

By day 70 of life, the patient was fully fed orally and no longer required gastric tube feeding. Periodic episodes of polyuria and vomiting persisted. He was fed a highly hydrolyzed infant formula; attempts to switch formulas led to increased vomiting. From the sixth week of life, oral sodium supplementation (10% NaCl) and subsequently potassium supplementation (Kalium syrup) were initiated. Hypernatremia observed just before discharge necessitated temporary discontinuation of oral sodium supplementation.

### Follow-up and long-term management

The patient has been followed for 30 months in the neonatology and nephrology outpatient clinics. Weight gain, developmental progress, and current therapy are summarized in Table 1, while laboratory results during follow-up are shown in Table 2.

**Table 1:** 30 months follow-up—treatment, diet, development and other problems.

Age	Weight [grams]	Treatment	Diet	Development	Additional data
3 months	3,280	Celecoxib 8 mg 2×/day Kalium 16 mEq/day Natrium 1 mEq/day IPP	Extensively hydrolyzed casein formula (Nutramigen) + 200 mL of water	Reduced muscle tension, establishes eye contact	BP 86/45 mmHg
6 months	4,650	Celecoxib 8 mg 2×/day Kalium 16 mEq/day	Extensively hydrolyzed casein formula (Nutramigen) + 250 mL water	Grasps toys, passes them from hand to hand, raising the head	In abdominal ultrasound echo-negative follicle, ursodeoxycholic acid was discontinued BP 90/58 mmHg
12 months	5,750	Reduction of celecoxib unsuccessful Continuation: Celecoxib 8 mg 2×/day Kalium 20 mEq/day	Extensively hydrolyzed casein formula (Nutramigen) strong thirst and desire to drink water	Reduced muscle tension, swivels, pivots, sitting independently, standing up with support	BP 100/50 mmHg
18 months	7,190	Celecoxib 8 mg 2×/day Kalium 20 mEq/day	Peptide-based, high-energy infant formula (Infantrini peptisorb) and 1.5 L of water, salting meals	Walks independently, contacts, says single words	Weight and height deficiency referred to endocrinology clinic BP 100/50 mmHg
24 months	8,000	Celecoxib 9 mg 2×/day Kalium 24 mEq/day	Peptide-based, high-energy infant formula (Infantrini peptisorb) and 2.5 L of water, salting meals	Progress in psychomotor development	Abundant diuresis, height and weight below the 3rd percentile BP 125/70 mmHg nephrocalcinosis—grade I b according to Hoyer
30 months	9,500	Celecoxib 11 mg 2×/day Kalium 30 mEq/day	Toddler's diet and 3–3.5 L of water, occasionally peptide-based, high-energy infant formula (Infantrini peptisorb), salting meals	Further motor development, speech development	Abundant diuresis, height and weight below the 3rd percentile BP 110/60 mmHg nephrocalcinosis—grade I b according to Hoyer, bilateral enlargement of the calyx-pelvic system enlarged ureters along their entire length

**Table 2:** 30 months follow-up—blood and urine samples results.

Age	3 months	6 months	12 months	18 months	24 months	30 months
Blood tests						
Na [mmol/L]	139	139	136	138.5	134.9	140.2
K [mmol/L]	4.48	4.04	4.1	3.6	5.31	4.05
P [mmol/L]	—	—	1.68	1.29	1.44	1.59
Mg[mmol/L]	—	—	0.94	1.01	—	0.95
Ca [mmol/L]	2.78	—	2.68	2.7	2.54	2.89
Creatinine [umol/L]	33	32	26	22.8	27.5	32.6
Uric acid [mmol/L]	—	—	264	231	—	191
Ht [%]	34.7	34.8	38.6	40.8	39.1	42.2
Hgb [g/dl]	11.6	11.7	12.8	14.2	13.5	14.8
Gasometry—capillary blood						
pH	7.46	7.44	7.42	7.47	7.36	7.44
HCO3 [mmol/L]	22.8	24.2	21.7	30	20.4	23.8
BE [mmol/L]	—0.8	0.4	2.1	5.7	—4.4	0
Urine samples						
Na [mmol/L]	69	—	<20	<20	—	<20
P [mmol/L]	—	3.53	3.1	—	—	2.91
K [mmol/L]	50.96	—	—	—	17.31	13.09
Creatinine [umol/L]	—	765	648	478	533	479
Uric acid [mmol/L]	—	773	526	—	294	373
Mg [mmol/L]	—	1.22	1.35	0.859	0.862	0.857
Ca [mmol/L]	2.61	2.33	2.21	1.97	1.9	2.15
Crystallisation indicators						
Ca/Crea	—	1.08	1.21	1.46	1.26	1.59
Mg/Crea	—	0.34	0.45	0.39	0.35	0.38
UA/Crea	—	1.51	1.21	—	0.82	1.16
P/Crea	—	1.26	1.31	0.57	0.51	1.66
Mg/Ca	—	0.32	0.37	0.26	0.28	0.24

In most cases, apart from episodes associated with infection, ionogram and blood gas parameters remain within normal limits. Renal function markers, including creatinine and uric acid levels, also fall within reference ranges, as do red blood cell indices in complete blood counts. However, with regard to crystallisation indices, elevated values are observed, particularly the calcium-to-creatinine ratio (Ca/Crea). The normal value for this age is <0.3. Exceeding this threshold may indicate hypercalciuria, which increases the risk of nephrocalcinosis and kidney stone formation.

**Figure 2:**
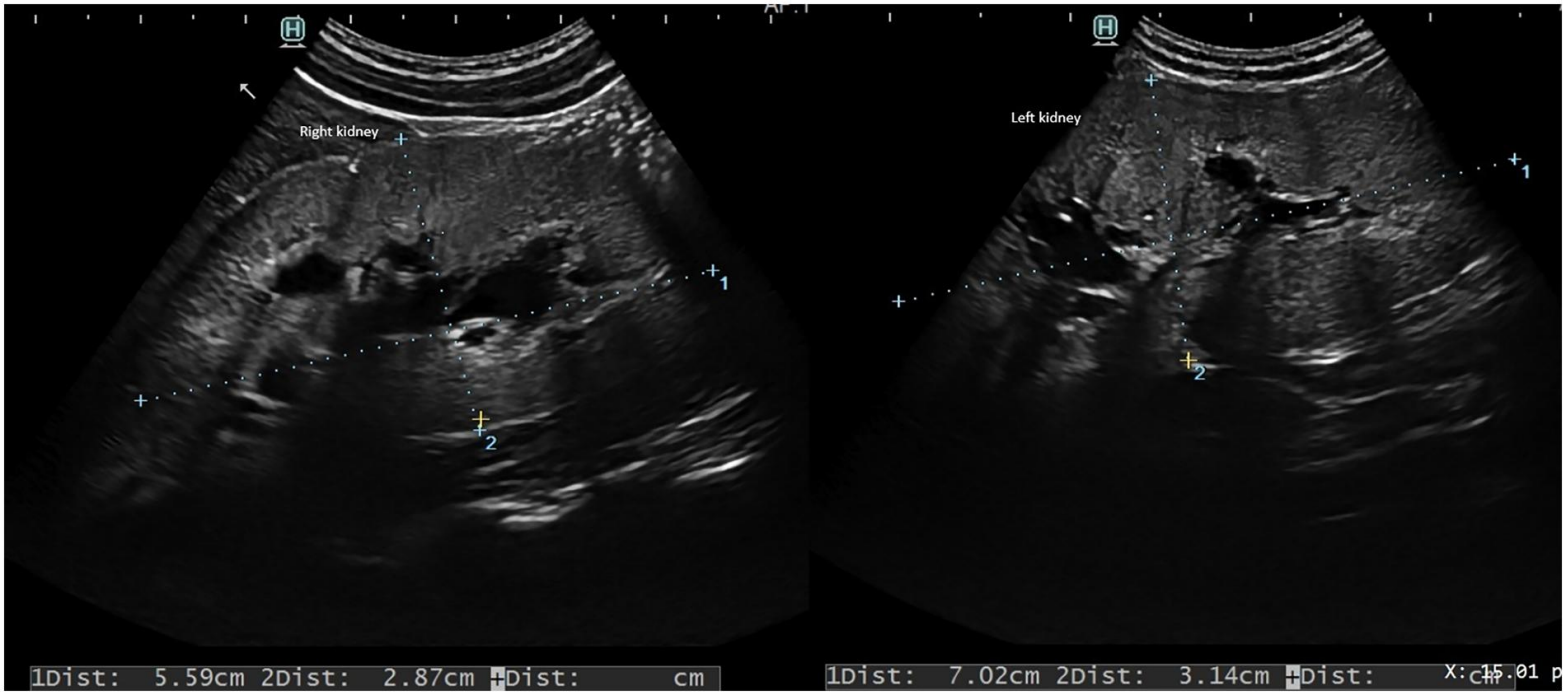
Left and right kidney: grade Ib nephrocalcinosis according to Hoyer, with hyperechoic margins of the renal pyramids, moreover bilateral enlargement of the calyx-pelvic system.

The patient consumed prescribed volumes of formula and supplemented fluid intake with mineral water. Over time, preference for water over formula led to suboptimal weight gain. By six months of age, gallbladder findings normalized and ursodeoxycholic acid therapy was discontinued. Renal ultrasonography, however, continues to demonstrate grade Ib nephrocalcinosis according to Hoyer, with hyperechoic margins of the renal pyramids, moreover bilateral enlargement of the calyx-pelvic system, enlarged ureters along their entire length [Figure 2].

Oral sodium supplementation has gradually been replaced by dietary salt, while potassium supplementation remains guided by serum ion monitoring. Attempts to reduce the celecoxib dose proved unsuccessful, as dose reduction led to disturbances in electrolyte balance, primarily hypokalemia. The patient is currently maintained on a regimen of approximately 1.1 mg/kg twice daily, which ensures stable and normal electrolyte levels.

Despite initial hypotonia, the patient demonstrates normal developmental progress with ongoing rehabilitation. Progression in the physical and intellectual development is observed.

Since 18 months of age, he has been under endocrinology supervision due to height and weight deficiencies. According to the WHO centile charts for corrected age, body weight remains below the 3rd centile.

To date, he has been hospitalised twice in the Paediatric Ward due to infections. At the age of 18 months, he was hospitalised because of pharyngitis accompanied by a reluctance to eat and at the age of 22 months on account of dehydration in the course of gastroenteritis caused by rotavirus infection. At the age of 30 months, he underwent outpatient treatment for gastroenteritis, but it was important to increase his oral potassium intake and monitor his ionogram frequently.

## Discussion

Bartter syndrome is a rare inherited tubulopathy characterized by hypokalemic metabolic alkalosis, polyuria, and salt wasting. In the presented case, the diagnosis was established promptly based on clinical presentation and confirmed by identification of a pathogenic variant in the *SLC12A1* gene. Early recognition allowed initiation of targeted management, including fluid and electrolyte replacement, which was essential for stabilization of renal function and correction of acid–base disturbances (14, 15).

Although the diagnostic process was straightforward, one of the most significant challenges in this patient's management was the discontinuation of parenteral nutrition. Due to persistent polyuria, high fluid and electrolyte requirements, gastrointestinal intolerance, gradual transition to full enteral feeding required careful adjustment of both enteral and parenteral support. Close monitoring of fluid and electrolyte management, combined with the off-label use of celecoxib, was essential to achieve euvolemia and maintain electrolyte homeostasis, ultimately allowing for the safe discontinuation of parenteral nutrition by day 61 of life.

Following stabilization, several long-term challenges emerged. Excessive thirst and preference for water over formula contributed to suboptimal weight gain. Despite careful nutritional management, growth remained a concern, necessitating endocrinology follow-up, and the patient might be considered for growth hormone therapy in the future. Renal ultrasonography revealed nephrocalcinosis, a well-recognized complication of Bartter syndrome resulting from chronic salt-wasting and elevated urinary calcium excretion. Although nephrocalcinosis did not impair renal function during follow-up, it necessitates ongoing surveillance. Currently, there is no specific therapy for hypercalciuria in Bartter syndrome. Therefore, long-term management relies on regular monitoring of serum electrolytes, vitamin D levels, and renal ultrasonography to assess for progression of nephrocalcinosis or development of nephrolithiasis. It is also important to remember that children with Bartter syndrome are particularly susceptible to rapid and significant electrolyte disturbances during even mild gastroenteritis, requiring close monitoring and prompt correction of fluid and electrolyte balance (10, 16–18).

Overall, the combination of careful fluid and electrolyte management, pharmacologic therapy with celecoxib, and supportive measures allowed stabilization of the patient's condition. This case highlights that, even when diagnosis is quick, achieving optimal nutritional support and managing the metabolic consequences of Bartter syndrome remain significant clinical challenges. Early expert consultation and individualized management are key to improving outcomes and minimizing complications.

## Summary

This case highlights that early recognition of type I Bartter syndrome allows prompt initiation of targeted therapy, but achieving stable fluid and electrolyte balance, establishing full enteral nutrition, and managing long-term complications such as nephrocalcinosis and growth impairment remain major challenges. Additionally, children with Bartter syndrome are particularly prone to rapid and significant electrolyte disturbances during even mild gastrointestinal infections, necessitating close monitoring and prompt correction of fluid and electrolyte losses.

## Data Availability

The original contributions presented in the study are included in the article/supplementary material, further inquiries can be directed to the corresponding author/s.
